# Osteoporosis and Related Health Status Among the Elderly Urban Residents in Elderly-Care Inns in Beijing, a Multicenter DXA Survey

**DOI:** 10.3389/fendo.2022.875678

**Published:** 2022-07-22

**Authors:** XinChao Lin, HongTao Guo, YiGang Lian, Jiajing Kou, GuangLei Wang, YiYun Chen, Juan Wang, Xu Han, Miao Jiang, QiaoHui Yang

**Affiliations:** ^1^ Dongzhimen Hospital, Beijing University of Chinese Medicine, Beijing, China; ^2^ The First Affiliated Hospital of Henan University of Chinese Medicine, Zhengzhou, China; ^3^ Institute of Basic Research in Clinical Medicine, China Academy of Chinese Medical Sciences, Beijing, China

**Keywords:** osteoporosis, osteopenia, DXA, urban area in Beijing, survey, risk factor

## Abstract

**Background:**

Identification of the high risk population for osteoporosis and timely prevention are the best strategies at present. Detailed epidemiological investigation in a well-defined population is necessary to explore the population-based characteristics and risk factors of osteoporosis, thus to facilitate better prevention programs.

**Method:**

In this prospective cross-sectional study, 1423 questionnaires were given out to the urban residents (female ≥ 40 years of age, male ≥50 years) who lived in the 27 Elderly-Care Inns interspersed among the seven central urban areas of Beijing. All participants were voluntary and underwent routine physical examination and spine and hip BMD measurements using the DXA instrument. The study protocols were approved by the Medical Ethics Committee of Dongzhimen Hospital, Beijing University of Chinese Medicine (JDZX2015079).

**Results:**

Altogether 1407 participants fulfilled the survey. Among 359 men, the prevalence of osteoporosis, osteopenia, and normal BMD were 18.1%, 56.6%, and 25.3%, respectively; among 1048 women, the corresponding figures were 40.3%, 42.8%, and 16.9%, respectively. After adjustment of age and BMI, both hands grip strength, height loss over 3 cm, serum levels of β-CTx, PINP, and OST were the independent risk factors for osteoporosis in both men and women; besides, familial Alzheimer’s disease history in men; and history of steatohepatitis and fracture, serum levels of PTH and ALT, age of menarche, age of menopause, and duration of menstruation in women were also risk factors of osteoporosis. In both genders, the cost-effective method, which adopted both hands grip strength, height loss over 3 cm, and medical history, indicated a good predictive ability to evaluate the risk of osteoporosis (in men AUC=0.730, 95%CI=0.642~0.817; in women AUC=0.769, 95%CI=0.724~0.813).

**Conclusions:**

In the population of elderly Beijing urban residents in Elderly-Care Inns, the prevalence of osteoporosis in women is higher than that in men and increases with aging more rapidly; the prevalence of osteopenia in men is higher than in women. The cost-effective method, including both hands grip strength, height loss over 3 cm, and familial Alzheimer’s disease history in men; fracture and steatohepatitis history as well as menstrual history in women is recommended in identifying the high-risk subjects for osteoporosis.

## Introduction

Osteoporosis is a kind of systemic bone disease characterized by decreased bone mass and bone microstructure damage, resulting in increased bone fragility and easy fracture (1). Osteopenia is a term to define bone density that is not normal but also not as low as osteoporosis (2). The prevalence rate of osteoporosis increases with age (3). In China, the prevalence rate of osteoporosis among the population above 60 years old was reported to be 36%; 23% in male population and 49% in female (4).

The most serious consequence of osteoporosis is osteoporotic fracture, which has been regarded as one of the most severe public health issues in middle-aged and elderly people concerned with the growth in the aging population throughout the world. It has been estimated that approximately 50% of women and 20% of men over 50 years old will suffer an osteoporosis-related fracture; hip fracture is the most devastating due to the consequent disability, mortality, and costs (5). The number of patients with osteoporosis fractures in China reached 2.33 million in 2010, including 360,000 hip fractures, 1.11 million vertebral fractures, and 860,000 other osteoporotic fractures (6). Among them, 20% of patients die of complications within one year after a hip fracture, and about 50% become disabled, with a significantly reduced quality of life (7). This brings a heavy economic burden on both the families and society, the medical expenditure of fractures in China in 2010 reached $10.2 billion USD, and this figure will be as high as $275 billion USD led by the estimated 5.99 million patients with osteoporotic fracture by the year 2050 (6).

Osteoporosis is preventable and treatable. Proper treatment can effectively reduce the risk of fracture and refracture even for those who have already suffered from brittle fracture (8). Thus, early screening and identification of high-risk populations so as to take timely preventive measures should never be over strengthened (9).

However, the fact is that osteoporosis is underdiagnosed and undertreated, especially in those over 75 years old, in whom treatment is probably most beneficial and cost-effective, and even among the highest-risk populations who have already suffered fractures in big cities such as Beijing (10), although many researchers and clinicians have already concentrated on these issues and made remarkable achievements, such as the publication of the guidelines for the diagnosis and treatment of osteoporosis both in modern medicine and in Chinese medicine (11–16).

Besides the efforts to improve the accessibility to bone densitometry, the awareness of the disease by professionals and the public, and the use and reimbursement of drugs, the first gap that needs to be closed in the structured care pathways for osteoporosis care is how to determine the risk factors so as to identify the high-risk subjects.

As a complex disease, osteoporosis is affected by multiple risk factors, including environmental factors and genetic factors. Genetic factors (uncontrollable factors), include race, aging, female menopause, and family history of brittle fracture. Environmental factors (controllable factors) include mainly unhealthy lifestyle such as smoking, alcohol consumption, nutritional imbalance, improper diet, low physical activity, and so on. These risk factors can individually or synergistically cause the loss of bone mineral density and lead to osteoporosis (17).

Notably, there exist significant differences in the lifestyle between different regions, countries, or ethnicities, for example, even the lifestyles between urban and rural areas and among different regions in China vary greatly, which leads to great differences in the prevalence rate of osteoporosis, the major risk factors, and the proper prevention measures among regions and populations (18).

The accurate and cost-effective clinical risk factor models for the assessment of osteoporosis probability should be based on the highly population-specific data. Unfortunately, work in this area is currently seriously insufficient in China, it has been pointed out that cost-effective analysis on screening strategy and intervention thresholds based on local epidemiology data and economic status are available only in Japan throughout the east Asia area (10). Therefore, the next key step should be focused on the establishment of local data to develop a cost-effective risk assessment strategy to identify high risk individuals for screening and treatment.

Regarding these considerations, the Chinese Society for Health Management of the Chinese Medical Association collaborated with the Chinese Geriatrics Society for Osteoporosis and Bone Mineral Research, for the first time, developed a multicenter epidemiological survey in a large-scale Chinese population for determining the prevalence of osteoporosis and establishing a BMD reference database with a unified DXA system (19).

This study was a sub-project of the above survey, undertaken by Dongzhimen Hospital, Beijing University of Chinese Medicine, the target population was about 1500 elderly urban residents in Beijing, China, in order to obtain the prevalence and risk factors of osteoporosis among them. The data from this survey were included in the first epidemiological study of osteoporosis among Chinese residents developed by the Chronic Disease Center of the Chinese Center for Disease Control and Prevention and the Osteoporosis and Bone Mineral Salt Diseases Branch of the Chinese Medical Association in 2018 organized by the National Health Commission.

## Materials and Methods

### Study Design and the Population

This was a prospective cross-sectional study. All participants were recruited from Beijing urban area and were voluntary and underwent routine physical examination carried out by physicians in the Health Management unnormal Center of Dongzhimen Hospital, Beijing University of Chinese Medicine.

Eligible participants must meet the inclusion criteria: female ≥ 40 years of age, male ≥50 years, and with spine and hip BMD measurements. Pregnant or lactating women; participants with a history of chronic diseases, such as metabolic bone disease, malignant tumors, calcium absorption; participants with acute infectious diseases; participants with mental health problems; and participants with a history of use of any drugs affecting bone metabolism were excluded. Each participant signed written informed consent.

The study protocols were approved by the Medical Ethics Committee of Dongzhimen Hospital, Beijing University of Chinese Medicine.(JDZX2015079). The registration number of the study was NCT02958020.

From July 26, 2017 to May 4, 2018, altogether 1423 questionnaires were given out to the middle-aged and elderly urban residents in Beijing, China. These residents were recruited from 27 Elderly-Care Inns interspersed among the seven central urban areas of Beijing. The distribution of the Elderly-Care Inns and corresponding population is shown in **Table 1**.

**Table 1:** Distribution of the recruited elderly-care inns in Beijing.

District		Elderly-Care Inns	Number	Total number
Dongcheng	1	Qianmen (前门驿站)	47	426
	2	Ganyu (甘雨驿站)	68	
	3	Wangfujing (王府井驿站)	17	
	4	Zhongyan (中央驿站)	92	
	5	Gulou (鼓楼驿站)	108	
	6	Shaojiu (韶九驿站)	69	
	7	Dongzhimen (东直门驿站)	25	
Chaoyang	1	Aoyuncun (奥运村驿站)	39	167
	2	Yanjingli (延静里驿站)	83	
	3	Dongdaqiao (东大桥驿站)	11	
	4	Kangying (康营驿站)	34	
Haidian	1	Fumeiyuan (福美苑驿站)	52	335
	2	Beixinjiayuan (北新家园驿站)	71	
	3	Diankeyuan (电科院驿站)	58	
	4	Yongtai (永泰庄驿站)	49	
	5	Jiancaixili (建材西里驿站)	88	
	6	Erlizhuang (二里庄驿站)	17	
Fengtai	1	Fangzhuang (方庄驿站)	41	127
	2	Xihongmen (西红门驿站)	86	
Daxing	1	Yizhuangzhangzhegongguan (亦庄长者公馆驿站)	30	30
Shijingshan	1	Yangzhuang (杨庄驿站)	134	266
	2	Tinan (体南驿站)	50	
	3	Taikang (泰康驿站)	33	
	4	Guchenggongyuan (古城公园驿站)	29	
	5	Baqianli (八千平驿站)	20	
Tongzhou	1	Tongzhouhuotun (通州霍屯)	38	72
	2	Xinhua (新华驿站)	34	
Total				1423

Among the 1423 distributed questionnaires, 11 informants were eliminated for improper age, two for incomplete information, three for unable to determine the TCM constitution type, finally 1407 participants were qualifiedly recruited.

### Data Collection

### Demographic Characteristics

Age, gender, address, height, weight, waistline, hipline, education level, marital status, occupation information, personal history (smoke, alcohol, dietary favor, pregnancy and parity history, menstrual history, etc.), medical history (chronic diseases, fracture history, medication use, sleep quality, etc.), were collected by specific designed form and face-to-face interviews.

### Questionnaires

International Physical Activity Scale (IPAQ) short form was applied to evaluate the physical activity (20); International Osteoporosis Foundation (IOF) was adopted for evaluating the osteoporosis risk (21); EuroQol Health Index Scale EQ-5D (EuroQol-5) dimension [17] was used to assess life quality. Questionnaires were carried out by physicians from the Health Management Center of Dongzhimen Hospital, Beijing University of Chinese Medicine.

### Blood Sample Collection

Fasting peripheral blood was collected from each participant. Levels of serum total calcium, serum phosphorus, serum magnesium, serum creatinine, alkaline phosphatase (ALP), thyrotropin (TPH), and 25-Hydroxyvitamin D were tested. Serum procollagen type 1 N-terminal propeptide (PINP), serum osteocalcin (OST), and β cross-linked C-telopeptide of type 1 collagen (β-CTx) were tested using radioimmunoassay. Blood samples were collected and tested by researchers from Guangzhou KingMed Diagnostics Group Co. Ltd.

### BMD Measurements and Osteoporosis Diagnosis

Each participant underwent a BMD measurement using the DXA instrument (Hologic, American Hologic WI), which was equipped in a BMD examination car, sponsored by Guangzhou KingMed Diagnostics Group Co. Ltd. The BMD examination car entered each community and provided BMD examination service to each participant, the lumbar spine (L1-L4) and the left proximal femur (Neck), the Wards triangle, the Troch, the Shaft, and the Total femur (Total) were measured.

Diagnostic criteria of osteoporosis were based on DXA bone mineral density T-score/Z-score (16). BMD values (g/cm^2^) were expressed as T- scores for men above 50 years old and postmenopausal women (number of SD above/below the mean for healthy 30-year-old adults); or as Z-scores for premenopausal women (number of SD above/below the mean for the patient’s age). The gender-specific mean BMD and standard deviation (SD) were calculated. The maximal gender-specific mean BMD was defined as the peak BMD. In accordance with the Chinese society of osteoporosis and mineral research (CSOBMR) criteria, osteoporosis was diagnosed using the following criteria:

**Table:** 

Normal:	T-score/Z-score ≥ -1.0 SD
Osteopenia:	-2.5 SD < T-score/Z-score<-1.0 SD
Osteoporosis:	T-score/Z-score≤ -2.5 SD

### Statistical Analysis

Continuous variables were expressed as mean ± SD and categorical variables as numbers (percentages). All analyses were stratified by gender. Descriptive statistics were performed using one-way analysis of variance and LSD methods were adopted in the *post hoc* test. For the variables that did not conform to the normal distribution, the Kruskal-Wallis H test was applied. Multivariate logistic regression analysis was conducted to evaluate the risk factors of osteoporosis including demographic variables, medical history, and physical and chemical examination variables. The interaction of age and BMI was included; OR value and 95%CI were calculated. The Youden’s index from the ROC was used to determine the cutoff values of the risk factors in predicting osteoporosis and osteopenia. A two-sided *P* value <0.05 was considered statistically significant. All analyses were conducted using SPSS24.0 software (IBM Corp., Armonk, NY, USA).

## Results

### Baseline Characteristics and Medical History of the Participants

The baseline characteristics of the participants are listed in **Table 2**. Altogether 1048 female and 359 male participants were involved in the analysis, the average age of men was 67.14 ± 7.44 (range from 50 to 87), of women was 64.33 ± 8.55 (range from 41 to 96). There were no significant differences among the three groups (normal, osteopenia, and osteoporosis population) in the distribution of monthly household income, working condition, history of smoke and alcohol intake in both genders, job category, and working condition in men.

**Table 2:** Baseline characteristics of study participants grouped by osteoporosis diagnose.

	Male						Female				
	Total	Normal	Osteopenia	Osteoporosis	*P* value		Total	Normal	Osteopenia	Osteoporosis	*P* value
Case number	359	91 (25.3%)	203 (56.6%)	65 (18.1%)			1048	177 (16.9%)	449 (42.8%)	422 (40.3%)	
Age category (in years)											
40-49	0	0	0	0	0.007*		55 (5.2%)	33 (60.0%)	21 (38.2%)	1 (1.8%)	<0.001*
50–59	64 (17.8%)	17 (26.6%)	38 (59.4%)	9 (14.1%)			299 (28.5%)	82 (27.4%)	137 (45.8%)	80 (26.8%)	
60–69	190 (52.9%)	49 (25.8%)	110 (57. 9%)	31 (16.3%)			467 (44.6%)	56 (12.0%)	210 (45.0%)	201 (43.0%)	
70–79	83 (23.1%)	23 (21.7%)	43 (51.8%)	17 (20.5%)			193 (18.4%)	6 (3.1%)	74 (38.3%)	113 (58.6%)	
80–89	22 (6.1%)	2 (9.1%)	12 (54.6%)	8 (36.4%)			32 (3.1%)	0	7 (21.9%)	25 (78.1%)	
≥90	0	0	0	0			2 (0.2%)	0	0	2 (100.0%)	
Monthly household income											
≤1000	18 (5.3%)	4 (22.2%)	11 (61.1%)	3 (16.7%)	0.672		53 (5.3%)	12 (22.6)	25 (47.2%)	16 (30.2%)	0.054
1000-3000	22 (6.5%)	5 (22.7%)	11 (50.0%)	6 (27.3%)			188 (18.7%)	38 (20.2%)	83 (44.1%)	67 (35.6%)	
3000-5000	110 (32.4%)	22 (20.0%)	64 (58.2%)	24 (21.8%)			326 (32.5%)	45 (13.8%)	141 (43.3%)	140 (42.9%)	
5000-8000	123 (36.2%)	32 (26.0%)	73 (59.3%)	18 (14.6%)			276 (27.5%)	54 (19.6%)	117 (42.4%)	105 (38.0%)	
8000-12000	51 (15.0%)	15 (29.4%)	27 (52.9%)	9 (17.6%)			113 (11.3%)	16 (14.2%)	39 (34.5%)	58(51.3%)	
12000-15000	6 (1.8%)	3 (50.0%)	2 (33.3%)	1 (16.7%)			30 (3.0%)	3 (10.0%)	17 (56.7%)	10 (33.3%)	
15000-20000	5 (1.5%)	3 (60.0%)	1 (20.0%)	1 (20.0%)			11 (1.1%)	3 (27.3%)	7 (63.6%)	1 (9.1%)	
≥20000	5 (1.5%)	2 (40.0%)	2 (40.0%)	1 (20.0%)			7 (0.7%)	0	2 (28.6%)	5 (71.4%)	
Job category											
Physical labor	129 (41.2%)	24 (18.6%)	78 (60.5%)	27 (20.9%)	0.168		363 (38.9%)	64 (17.6%)	145 (39.9%)	154 (42.4%)	0.011*
Mental labor	86 (27.5%)	25(29.1%)	48 (55.8%)	13 (15.1%)			305 (32.7%)	36 (11.9%)	127 (41.9%)	140 (46.2%)	
Both physical and mental labor	98 (31.3%)	31 (31.6%)	53 (54.1%)	14 (14.3%)			267 (28.6%)	59 (22.1%)	118 (44.2%)	90 (33.7%)	
Working condition											
keep working	23 (6.6%)	8 (34.8%)	13 (56.5%)	2 (8.7%)	0.350		88 (8.7%)	31 (35.2%)	36 (40.9%)	21 (23.9%)	<0.001*
Retired	328 (93.4%)	80 (24.4%)	187 (57.10%)	61 (18.6%)			929 (91.3%)	145 (15.6%)	398 (42.8%)	386 (41.6%)	
Education level											
Illiteracy	3 (0.8%)	0	3 (100.0%)	0	0.019*		32 (3.1%)	1 (3.1%)	15 (46.9%)	16 (50.0%)	<0.001*
Primary school	17 (4.8%)	4 (23.5%)	8 (47.1%)	5 (29.4%)			88 (8.4%)	6 (6.8%)	31 (35.2%)	51 (58.0%)	
Junior high school	135 (38.2%)	24 (18.0%)	77 (57.9%)	32 (24.1%)			336 (32.2%)	42 (12.5%)	146 (43.5%)	148 (44.0%)	
High school	108 (30.6%	34 (31.5%)	65 (6.02%)	9 (8.3%)			364 (34.9%)	84 (23.1%)	159 (43.7%)	121 (33.2%)	
College	59 (16.7%)	15 (25.4%)	34 (57.6%)	10 (16.9%)			142 (13.6%)	28 (19.9%)	60 (42.6%)	53 (37.6%)	
Bachelor degree or above	33 (9.3%)	13 (39.4%)	13 (39.4%)	7 (21.2%)			82 (7.9%)	16 (19.5%)	35 (42.7%)	31 (37.8%)	
Marriage											
Married	317 (90.3%)	80 (25.2%)	182 (57.4%)	55 (17.4%)	0.043*		841 (80.9%)	162 (19.3%)	364 (43.3%)	315 (37.5%)	<0.001*
Single	3 (0.9%)	1 (33.3%)	0	2 (66.7%)			8 (0.8%)	1 (12.5%)	3 (37.5%)	4 (50.0%)	
Divorced	13 (3.7%)	1 (7.7%)	8 (61.5%)	4 (30.8%)			36 (3.5%)	6 (16.7%)	17 (47.2%)	13 (36.1%)	
Widowed	18 (5.1%)	8 (44.4%)	9 (50.0%)	1 (5.6%)			154 (14.8%)	8 (5.2%)	57 (37.0%)	89 (57.8%)	
Smoke											
No smoking	142 (40.1%)	45 (31.7%)	71 (50.0%)	26 (18.3%)	0.248		985 (95.3%)	165 (16.8%)	420 (42.6%)	400 (40.6%)	0.976
Active smoking	126 (35.6%)	27 (21.4%)	75 (59.5%)	24 (19.0%)			37 (3.6%)	7 (18.9%)	16 (43.2%)	14 (37.8%)	
Passive smoking	86 (24.3%)	18 (20.9%)	53 (61.6%)	15 (17.4%)			12 (1.1%)	2 (16.7%)	6 (50.0%)	4 (33.3%)	
History of alcohol intake											
No alcohol use	141 (40.8%)	40 (28.4%)	73 (51.8%)	28 (19.9%)	0.739		962 (93.3%)	162 (16.8%)	404 (42.0%)	396 (41.2%)	0.129
Alcohol drinking	171 (48.0%)	41 (24.0%)	102 (59.6%)	28 (16.4%)			57 (5.5%)	12 (21.1%)	29 (50.9%)	16 (28.1%)	
Drinking in the past	40 (11.2%)	10 (25.0%)	23 (57.5%)	7 (17.5%)			12 (1.2%)	2 (16.7%)	8 (66.7%)	2 (16.7%)	

Values are presented as N (%). P < 0.05 stands for a significant difference among the three groups (Normal, Osteopenia, and Osteoporosis).

Medical history of study participants and related medications were collected, including fracture, cerebral infarction, coronary heart disease, hyperlipidemia, hypertension, lumbar intervertebral disc protrusion, lumbar spinal stenosis, knee osteoarthritis, gout, gastritis, gastric ulcer, type 2 diabetes, steatohepatitis, chronic pharyngolaryngitis, allergic rhinitis, cerebral hemorrhage, hyperthyreosis, hypothyroidism, hyperparathyroidism, chronic nephrosis, diabetic nephropathy, kidney transplantation, type 1 diabetes, chronic hepatitis A, chronic hepatitis B, liver cirrhosis, liver transplantation, alcoholic liver disease, breast cancer, liver cancer, ovarian cancer, thyroid cancer, chronic bronchitis, pulmonary emphysema, chronic obstructive pulmonary disease (COPD), bronchial asthma, bronchiectasia, and pulmonary interstitial fibrosis. **Table 3** lists the prevalence rates and inter group differences of 15 diseases with high prevalence. In female population, the prevalence of fracture, coronary heart disease, and hypertension increased as BMD decreased (^*^P<0.05); the prevalence of steatohepatitis showed significant difference among the three groups (P<0.05). There was no significant difference of the disease history distribution among the three groups in men (^*^P < 0.05).

**Table 3:** Medical history of study participants.

Disease	Male				Female			
	Normal(91)	Osteopenia(203)	Osteoporosis(65)	*P* value	Normal(177)	Osteopenia(449)	Osteoporosis(422)	*P* value
Fracture	14 (18.7%)	44 (58.7%)	17 (22.7%)	0.215	31 (13.6%)	79 (34.6%)	118 (51.8%)	<0.001*
Cerebral infarction	3 (8.8%)	22 (64.7%)	9 (26.5%)	0.051	6 (11.5%)	17 (32.7%)	29 (55.8%)	0.064
Coronary heart disease	19 (30.2%)	28 (44.4%)	16 (25.4%)	0.085	14 (9.3%)	64 (42.4%)	73 (48.3%)	0.012*
Hyperlipidemia	31 (29.5%)	60 (57.1%)	14 (13.3%)	0.235	52 (13.5%)	170 (44.3%)	162 (42.2%)	0.088
Hypertension	43 (25.1%)	97 (56.7%)	31 (18.1%)	0.870	59 (12.8%)	201 (43.7%)	200 (43.5%)	0.006*
Lumbar intervertebral disc protrusion	12 (20.3%)	37 (62.7%)	10 (16.9%)	0.542	43 (20.0%)	99 (46.0%)	73 (34.0%)	0.087
Lumbar spinal stenosis	6 (20.0%)	16 (53.3%)	8 (26.7%)	0.416	22 (19.3%)	50 (43.9%)	42 (36.8%)	0.656
Knee osteoarthritis	7 (17.1%)	26 (63.4%)	8 (19.5%)	0.430	38 (15.8%)	109 (45.4%)	93 (38.8%)	0.649
Gout	8 (47.1%)	7 (41.2%)	2 (11.8%)	0.108	4 (16.7%)	13 (54.2%)	7 (29.2%)	0.475
Gastritis	9 (22.0%)	24 (58.5%)	8 (19.5%)	0.863	27 (17.4%)	64 (41.3%)	64 (41.3%)	0.914
Gastric ulcer	5 (22.7%)	14 (63.6%)	3 (13.6%)	0.767	4 (12.9%)	8 (25.8%)	19 (61.3%)	0.051
Type 2 diabetes	24 (29.3%)	49 (59.8%)	9 (11.0%)	0.148	24 (13.5%)	75 (42.1%)	79 (44.4%)	0.301
Steatohepatitis	25 (34.2%)	39 (53.4%)	9 (12.3%)	0.095	42 (19.9%)	106 (50.2%)	63 (29.9%)	0.003*
Chronic pharyngolaryngitis	19 (27.1%)	37 (52.9%)	14 (20.0%)	0.782	54 (19.1%)	119 (42.2%)	109 (38.7%)	0.483
Allergic rhinitis	20 (30.3%)	38 (57.6%)	8 (12.1%)	0.288	26 (20.3%)	62 (48.4%)	40 (31.3%)	0.081

Values are presented as N (%).

P<0.05 stands for a significant difference among groups (Normal, Osteopenia, and Osteoporosis) adjusted by age and BMI.

^*^P<0.05.

Among the three groups, the distributions of age, education level, and marital status in both genders; job category and working condition in women, showed statistical differences (*P*<0.05). Subjects with elder age, physical worker, single status, and those with lower education levels tended to have lower BMD.

### The Prevalence Rates of Osteopenia and Osteoporosis in the Participants

**Figure 1:**
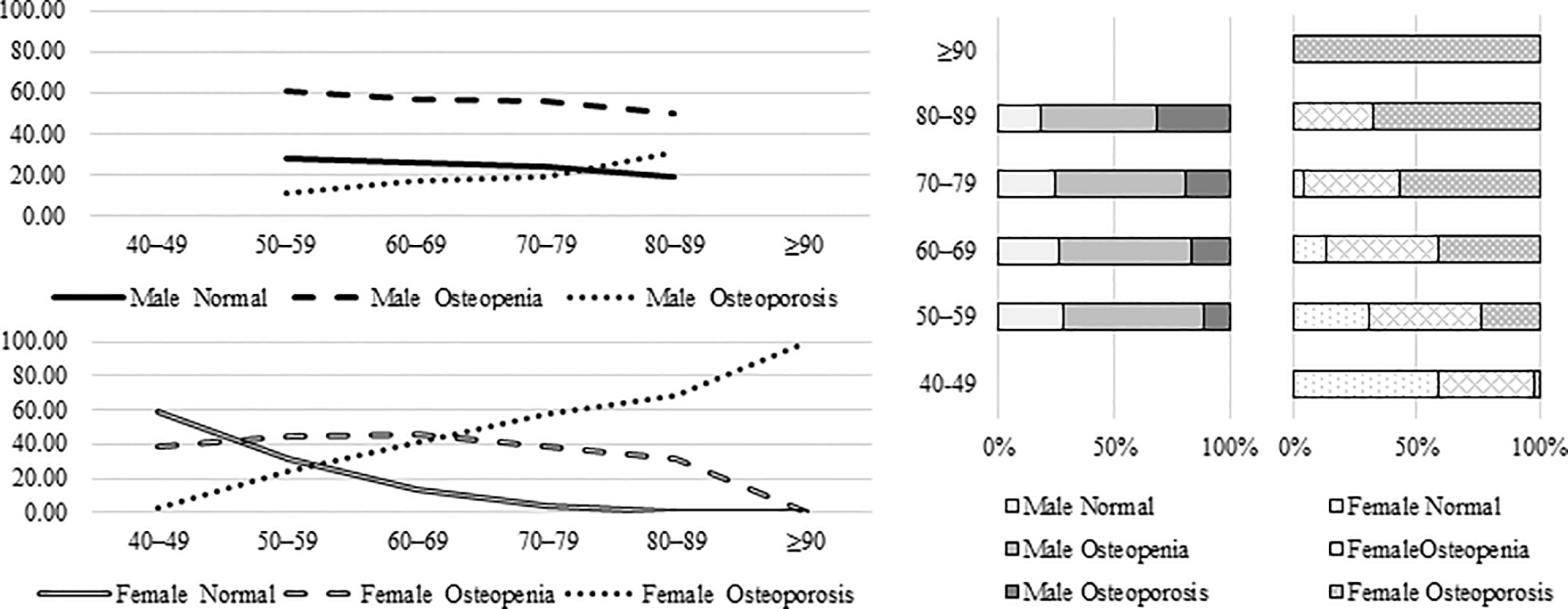
The prevalence of osteoporosis in the participants by age and gender.

The overall prevalence rate of osteoporosis was 34.6% and osteopenia was 46.3% as listed in **Table 4**. In male population, the prevalence of osteoporosis and osteopenia was 18.1% and 56.6%, respectively; in the female population it was 40.3% and 42.8%, respectively. The prevalence of both osteoporosis and osteopenia in senile women were much higher than those in senile men. **Figure 1** shows the prevalence of osteoporosis and osteopenia by age and gender. With the increase of age, the prevalence of osteoporosis elevates more significantly in women.

**Table 4:** The prevalence rates of osteoporosis in participants by gender.

	Total	Male	Female
	n=1407	n=359	n=1048
Normal	268 (25.6%)	91 (25.3%)	177 (16.9%)
Osteopenia	652 (46.3%)	203 (56.6%)	449 (42.8%)
Osteoporosis	487 (34.6%)	65 (18.1%)	422 (40.3%)

After the age of 50, the prevalence of osteoporosis in men increased slowly, but the prevalence of osteopenia was always higher than that in women in the same age period. Therefore, the issue of BMD declining in elderly men also needs to be paid high attention. In women, the prevalence of osteoporosis after the age of 50 increased quickly with age. After 80 years old, all women indicated abnormal BMD; the prevalence of osteopenia kept relatively stable. The characteristics of BMD decline between men and women were different.

### Risk Factors for Osteoporosis Analyses

The results of physical examination, biochemical test, and bone metabolic examination are listed in **Table 5**.

**Table 5:** Physical and chemical indexes by gender and osteoporosis diagnose.

	**Total**	**Normal**	**Osteopenia**	**Osteoporosis**	***P* value**	***P* value by LSD**
**Male (N=364)**						
Height (cm)	170.01 ± 5.80	171.79 ± 5.70	170.33 ± 5.38	166.77 ± 5.95	<0.001*	0.038@, <0.001#, <0.001$
Weight (kg)	73.15 ± 11.14	75.07 ± 14.81	73.97 ± 9.36	67.89 ± 8.56	<0.001*	<0.001#, <0.001$
BMI (kg/cm^2^)	25.29 ± 3.52	25.48 ± 4.90	25.48 ± 2.93	24.41 ± 2.77	0.083	
Waistline (cm)	90.15 ± 16.80	92.81 ± 15.74	89.85 ± 18.23	87.27 ± 12.60	0.128	
Hipline (cm)	99.38 ± 16.83	102.37 ± 15.35	98.51 ± 18.30	97.89 ± 13.17	0.148	
W/H	0.91 ± 0.08	0.91 ± 0.07	0.91 ± 0.09	0.89 ± 0.06	0.211	
Grip strength of left hand (Kg)	32.73 ± 8.88	36.04 ± 9.68	31.82 ± 8.42	31.01 ± 8.03	<0.001*	<0.001@, <0.001#
Grip strength of right hand (Kg)	34.35 ± 8.67	37.35 ± 7.55	33.51 ± 8.99	32.77 ± 8.21	0.001*	<0.001@, 0.001#
Sit test (S)	9.30 ± 4.26	8.63 ± 2.76	9.48 ± 3.73	9.65 ± 6.75	0.050	
Alkaline phosphatase-ALP (U/L)	72.74 ± 22.33	68.60 ± 16.06	74.29 ± 24.68	73.70 ± 21.66	0.126	
Creatinine (μmol/L)	82.14 ± 15.25	84.45 ± 14.20	81.34 ± 14.13	81.40 ± 19.46	0.255	
Parathyroid hormone-PTH (pmol/L)	3.10 ± 1.21	3.01 ± 1.13	3.06 ± 1.23	3.34 ± 1.23	0.191	
Osteocalcin-OST (ng/mL)	10.75 ± 3.88	9.74 ± 3.19	10.78 ± 3.93	12.10 ± 4.21	0.001*	0.034@, <0.001#, 0.017$
Serum Phosphorus (mol/L)	1.23 ± 0.22	1.22 ± 0.23	1.23 ± 0.23	1.22 ± 0.21	0.927	
Serum Magnesium (mmol/L)	0.93 ± 0.80	0.93 ± 0.80	0.93 ± 0.82	0.94 ± 0.72	0.598	
Serum Total Calcium (mmol/L)	2.35 ± 0.85	2.36 ± 0.08	2.35 ± 0.09	2.34 ± 0.08	0.271	
β-CTx (ng/mL)	0.24 ± 0.11	0.20 ± 0.08	0.24 ± 0.11	0.28 ± 0.12	<0.001*	0.004@, <0.001#, 0.017$
PINP (ng/mL)	38.44 ± 15.05	33.97 ± 13.87	39.23 ± 15.04	42.22 ± 15.44	0.002*	0.006@, 0.001#
25-hydroxyvitamin D3 (ng/mL)	15.41 ± 5.78	16.31 ± 5.97	15.10 ± 5.65	15.20 ± 5.83	0.231	
25-hydroxyvitamin D2 (ng/mL)	3.49 ± 2.88	4.06 ± 3.36	3.51 ± 2.97	2.54 ± 1.24	0.304	
25-hydroxyvitamin D Total (ng/mL)	16.20 ± 6.04	17.32 ± 6.32	15.85 ± 5.93	15.77 ± 5.86	0.304	
**Female (N=1055)**						
Height (cm)	158.72 ± 5.64	160.53 ± 6.24	159.28 ± 5.29	157.36 ± 5.43	<0.001*	0.011@, <0.001#, <0.001$
Weight (kg)	63.09 ± 9.52	68.60 ± 9.50	64.14 ± 9.22	59.65 ± 8.46	<0.001*	<0.001@, <0.001#, <0.001$
BMI (kg/cm^2^)	25.04 ± 3.56	26.64 ± 3.67	25.30 ± 3.57	24.09 ± 3.20	<0.001*	<0.001@, <0.001#, <0.001$
Waistline (cm)	86.38 ± 52.53	87.28 ± 66.47	85.97 ± 39.14	86.45 ± 58.07	0.961	
Hipline (cm)	96.75 ± 18.97	96.30 ± 21.00	97.84 ± 20.42	95.77 ± 16.27	0.262	
W/H	0.89 ± 0.49	0.90 ± 0.59	0.88 ± 0.37	0.90 ± 0.55	0.830	
Grip strength of left hand (Kg)	22.35 ± 6.92	25.01 ± 6.58	22.88 ± 7.67	20.68 ± 5.67	<0.001*	<0.001@, <0.001#, <0.001$
Grip strength of right hand (Kg)	23.22 ± 6.02	25.57 ± 6.10	23.51 ± 5.65	21.92 ± 6.04	<0.001*	<0.001@, <0.001#, <0.001$
Sit test (S)	8.95 ± 3.57	8.17 ± 3.33	8.66 ± 2.89	9.61 ± 4.18	<0.001*	0.006@, <0.001#, 0.003$
Alkaline phosphatase-ALP (U/L)	78.75 ± 23.45	69.97 ± 20.33	77.88 ± 21.98	83.45 ± 25.01	<0.001*	<0.001@, <0.001#, 0.001$
Creatinine (μmol/L)	62.95 ± 11.72	61.30 ± 9.63	63.12 ± 10.52	63.47 ± 11.72	0.114	
Parathyroid hormone-PTH (pmol/L)	3.41 ± 1.37	3.18 ± 1.24	3.37 ± 1.29	3.55 ± 1.48	0.009*	0.003#
Osteocalcin-OST (ng/mL)	14.18 ± 5.15	11.51 ± 3.60	13.93 ± 4.69	15.63 ± 5.67	<0.001*	<0.001@, <0.001#, <0.001$
Serum Phosphorus (mol/L)	1.36 ± 0.21	1.36 ± 0.21	1.36 ± 0.20	1.35 ± 0.22	0.882	
Serum Magnesium (mmol/L)	0.93 ± 0.07	0.91 ± 0.07	0.93 ± 0.07	0.93 ± 0.08	0.019*	0.015@, 0.007#
Serum Total Calcium (mmol/L)	2.37 ± 0.08	2.37 ± 0.09	2.37 ± 0.08	2.37 ± 0.09	0.981	
β-CTx (ng/mL)	0.29 ± 0.12	0.23 ± 0.11	0.28 ± 0.11	0.32 ± 0.13	<0.001*	<0.001@, <0.001#, <0.001$
PINP (ng/mL)	48.73 ± 19.97	41.60 ± 14.71	48.77 ± 18.73	51.80 ± 22.35	<0.001*	<0.001@, <0.001#, 0.047$
25-hydroxyvitamin D3 (ng/mL)	14.66 ± 5.77	14.89 ± 5.87	14.61 ± 5.56	14.60 ± 5.95	0.178	
25-hydroxyvitamin D2 (ng/mL)	4.09 ± 4.81	4.90 ± 6.56	3.66 ± 3.71	4.29 ± 5.18	0.364	
25-hydroxyvitamin D Total (ng/mL)	15.68 ± 6.25	15.85 ± 6.50	15.57 ± 6.05	15.73 ± 6.37	0.871	

values are presented as mean ± SD.

*: P<0.05 among the 3 groups (Normal, Osteopenia, and Osteoporosis) by ANOVA.

@: P<0.05 between the Normal group and the Osteopenia group;

#: P<0.05 between the Normal group and the Osteoporosis group;

$: P<0.05 between the Osteopenia group and the Osteoporosis group.

β-CTx stands for β cross-linked C-telopeptide of type 1 collagen; PINP represented Procollagen type 1 N-terminal propeptide.

The male case number involved in the tests of β-CTx, PINP, 25-hydroxyvitamin D3, and 25-hydroxyvitamin D Total was 357; and in the test of 25-hydroxyvitamin D2 is 80; The female case numbers in the above two parts are 1012 and 252, respectively.

TCM symptoms and constitution differentiation, International Physical Activity Scale (IPAQ) short form, International Osteoporosis Foundation (IOF), and EuroQol Health Index Scale EQ-5D (EuroQol-5) were also adopted and the information were analyzed, since no positive result was detected, the relative results were ignored.

Univariate analysis showed that in the male population, height, weight, grip strength of each hand, OST, β-CTx, and PINP presented significant differences among groups (*P*<0.05), the *P* values in post analysis by LSD method are listed as well. In the female population, height, weight, BMI, grip strength of each hand, sit test, ALP, PTH, OST, serum magnesium, β-CTx, and PINP show differences among groups (*P*<0.05).

### Risk Factors Analysis and the Predictive Ability for Osteoporosis

Multivariate analysis was adopted to determine the risk factors of osteoporosis and the results are displayed and ordered by OR value in **Table 6**. For men, β-CTx, familial Alzheimer disease, OST, height loss, and PINP were risk factors for osteoporosis after adjustment of age and BMI. Grip strength of each hand showed slight negative correlation with osteoporosis. Men with familial Alzheimer’s disease history, height loss (>3cm), and higher levers of serum β-CTx, PINP, OST, and lower hands grip strength had a higher risk of osteoporosis. More risk factors were screened in women after adjustment of age and BMI, including β-CTx, fracture history, height loss (>3cm), PTH, OST, age of menarche, ALP, PINP; while grip strength of each hand, age of menopause, duration of menstruation, and steatohepatitis history were negatively correlated with osteoporosis.

**Table 6:** Multivariate analysis of relevant risk factors for osteoporosis.

Variables	B regression coefficient	*P* value	Exp(B)/OR	95%CI
**Male**				
β-CTx (ng/mL)	4.380	<0.001	79.838	10.602~601.845
Familial Alzheimer Disease	1.020	0.033	2.773	1.087~7.078
Osteocalcin-OST (ng/mL)	0.96	0.01	2.612	1.042~1.162
Height loss (>3cm)	0.478	0.037	1.613	1.028~2.527
PINP (ng/mL)	0.25	0.001	1.284	1.011~1.040
Grip strength of left hand (Kg)	-0.040	0.002	0.961	0.936~0.986
Grip strength of right hand (Kg)	-0.036	0.008	0.965	0.939~0.990
**Female**				
β-CTx (ng/mL)	3.736	<0.001	41.93	14.397~122.120
Fracture	0.412	0.007	1.510	1.116~2.042
Height loss (>3cm)	0.310	0.024	1.363	1.042~1.786
Parathyroid hormone-PTH (pmol/L)	0.168	<0.001	1.183	1.087~1.307
Osteocalcin-OST (ng/mL)	0.100	<0.001	1.105	1.077~1.134
Age of menarche (Year)	0.085	0.009	1.089	1.021~1.161
Alkaline phosphatase-ALP (U/L)	0.019	<0.001	1.019	1.013~1.025
PINP (ng/mL)	0.018	<0.001	1.018	1.116~1.025
Grip strength of right hand (Kg)	-0.029	0.010	0.971	0.950~0.993
Grip strength of left hand (Kg)	-0.032	0.001	0.969	0.951~0.987
Age of menopause (Year)	-0.048	0.002	0.953	0.924~0.982
Duration of menstruation (Day)	-0.072	0.039-	0.931	0.870~0.996
Steatohepatitis	-0.390	0.013	0.677	0.498~0.922

All variables were analyzed after adjustment by age and BMI. β-CTx stood for β cross-linked C-telopeptide of type 1 collagen; PINP represented Procollagen type 1 N-terminal propeptide. The cutoff value was calculated and the ROC curve was drawn of each selected risk factor to determine the predictive ability for osteoporosis. The sensitivity, specificity, and the AUC (area under the curve) with 95%CI, and P value for predicting osteoporosis are displayed in **Table 7**.

In both men and women, the AUC of one single factor ranged from 0.55 to 0.75. To achieve better AUC, the factors were grouped and combined together. One group was cost-effective group, including both hands grip strength and medical history (including family history, decrease of body height (>3cm), chronic disease, and menstrual history in women); the other group was serum test group (including serum levels of β-CTx, PINP, and OST in men and β-CTx, PINP, OST, ALP, and PTH in women). Finally, the two groups were combined together as one predictor. The best predictive ability was achieved by the final combined predictor in both men and women, which reached 79.7% in men and 82.6% in women. Notably, the predictive ability of the cost-effective group for osteoporosis was also desirable, and reached 73.0% in men and 76.9% in women, respectively. Both were higher than the predictive ability of serum test group. The predictive ability of these factors for abnormal BMD (including both osteopenia and osteoporosis) was similar to, and a little lower than, that for osteoporosis.

**Table 7:** Predictive ability of the risk factors for predicting osteoporosis and osteopenia by gender.

Variables	Disease for predicting	Optimal cutoffs	Sensitivity	Specificity	AUC	95%CI	*P* value	
**Male**									
β-CTx (ng/mL)	Osteoporosis	0.20	**0.794**	0.595	**0.714**	0.630~0.798	<0.001	
	Osteopenia +osteoporosis	0.21	0.620	0.640	0.636	0.570~0.702	<0.001	
PINP (ng/mL)	Osteoporosis	28.97	0.841	0.494	0.671	0.583~0.759	<0.001	
	Osteopenia +osteoporosis	28.90	**0.741**	0.494	0.634	0.565~0.703	<0.001	
Osteocalcin-OST (ng/mL)	Osteoporosis	13.65	0.381	0.921	0.677	0.591~0.763	<0.001	
	Osteopenia +osteoporosis	7.35	0.867	0.303	0.598	0.531~0.665	0.006	
Grip strength of left hand (Kg)	Osteoporosis	33.80	0.640	0.675	0.671	0.548~0.758	<0.001	
	Osteopenia +osteoporosis	33.55	0.625	0.622	0.648	0.584~0.713	<0.001	
Grip strength of right hand (Kg)	Osteoporosis	35.45	0.640	0.625	0.660	0.573~0.748	0.001	
	Osteopenia +osteoporosis	32.55	0.764	0.472	0.640	0.576~0.704	<0.001	
β-CTx+ PINP+ OST	Osteoporosis	–	–	–	**0.708**	0.623~0.792	<0.001	
	Osteopenia +osteoporosis	–	–	–	0.649	0.583~0.715	<0.001	
Familial Alzheimer Disease+ Height loss (>3cm) + Both Hand Grip strength	Osteoporosis	–	–	–	**0.730**	0.642~0.817	<0.001	
	Osteopenia +osteoporosis	–	–	–	0.672	0.600~0.743	<0.001	
Familial Alzheimer Disease+ Height loss (>3cm) + Both Hand Grip strength+β-CTx+ PINP+ OST	Osteoporosis	–	–	–	**0.797**	0.715~0.879	<0.001	
	Osteopenia +osteoporosis	–	–	–	**0.717**	0.652~0.780	<0.001	
**Female**									
β-CTx (ng/mL)	Osteoporosis	0.27	0.591	**0.743**	**0.707**	0.661~0.752	<0.001	
	Osteopenia +osteoporosis	0.27	0.533	**0.739**	0.675	0.632~0.718	<0.001	
Parathyroid hormone-PTH (pmol/L)	Osteoporosis	3.68	0.369	**0.754**	0.573	0.523~0.623	0.005	
	Osteopenia +osteoporosis	3.67	0.349	**0.750**	0.552	0.507~0.598	0.029	
Osteocalcin-OST (ng/mL)	Osteoporosis	14.66	0.529	0.840	**0.731**	0.688~0.773	<0.001	
	Osteopenia +osteoporosis	14.53	0.472	0.830	0.691	0.651~0.732	<0.001	
Age of menarche (Year)	Osteoporosis	13.50	**0.718**	0.454	0.613	0.563~0.663	<0.001	
	Osteopenia +osteoporosis	13.50	0.684	0.451	0.581	0.534~0.627	0.001	
Alkaline phosphatase-ALP (U/L)	Osteoporosis	71.50	0.663	0.640	0.682	0.635~0.729	<0.001	
	Osteopenia +osteoporosis	71.50	0.613	0.642	0.656	0.611~0.700	<0.001	
PINP (ng/mL)	Osteoporosis	37.13	**0.768**	0.474	0.652	0.605~0.700	<0.001	
	Osteopenia +osteoporosis	38.16	**0.724**	0.500	0.636	0.592~0.680	<0.001	
Grip strength of left hand (Kg)	Osteoporosis	19.91	0.864	0.459	**0.709**	0.664~0.753	<0.001	
	Osteopenia +osteoporosis	24.05	0.599	0.695	0.659	0.612~0.707	<0.001	
Grip strength of right hand (Kg)	Osteoporosis	22.25	**0.780**	0.521	0.689	0.643~0.735	<0.001	
	Osteopenia +osteoporosis	22.65	**0.743**	0.487	0.626	0.576~0.677	<0.001	
Age of menopause (Year)	Osteoporosis	49.50	**0.764**	0.420	0.627	0.575~0.680	<0.001	
	Osteopenia +osteoporosis	49.50	**0.757**	0.400	0.612	0.561~0.662	<0.001	
Duration of menstruation (Day)	Osteoporosis	4.50	0.861	0.274	0.565	0.516~0.614	0.013	
	Osteopenia +osteoporosis	–	–	–	–	–	–	
β-CTx+ PINP+ OST+ ALP+ PTH	Osteoporosis	–	–	–	**0.760**	0.719~0.801	<0.001	
	Osteopenia +osteoporosis	–	–	–	**0.716**	0.676~0.757	<0.001	
Fracture+ Height loss (>3cm) + Steatohepatitis+ hands Grip strength + Age of menarche+ Age of menopause +Duration of menstruation	Osteoporosis	–	–	–	**0.769**	0.724~0.813	<0.001	
	Osteopenia +osteoporosis	–	–	–	0.692	0.646~0.738	<0.001	
β-CTx+ PINP+ OST+ ALP+ PTH + Fracture+ Height loss (>3cm) + Steatohepatitis+ hands Grip strength + Age of menarche+ Age of menopause +Duration of menstruation	Osteoporosis	–	–	–	**0.826**	0.787~0.864	<0.001	
	Osteopenia +osteoporosis	–	–	–	**0.753**	0.711~0.795	<0.001	

All variables are analyzed after adjustment by age and BMI. β-CTx stands for β cross-linked C-telopeptide of type 1 collagen; PINP represents Procollagen type 1 N-terminal propeptide.

The bold type is used when the AUC value > 0.7.

## Discussion

To our best knowledge, this is the first epidemiological investigation for osteoporosis based on a well-defined population in the Beijing urban area. In this group of subjects, it has been revealed that the cost-effective method could be used as a predictive factor for early identification of high-risk subjects for osteoporosis and osteopenia.

### The Significance of Population Selection in This Study

Previous studies usually concentrated on a certain group of subjects, such as the elderly (22), postmenopausal women (23, 24), persons with physical disabilities (25), or people in some region or a country (26–28). These studies facilitated to deepen our understanding of the role of human factors in the development of osteoporosis and promoted the guidelines for the prevention and treatment of osteoporosis. However, the controllable factors, or lifestyle factors, should receive more attention because they also play an undeniably important role on the occurrence of osteoporosis. The diagnosis and treatment rate of osteoporosis still varied significantly among regions and between urban and rural areas, therefore, epidemiological investigation of osteoporosis in specific populations with similar lifestyles in the same living area could help to find more effective prevention and treatment methods for this population.

Aging and urbanization have been the two major issues in China, which make lifestyle changes significant, especially in the big cities such as Beijing, Shanghai, and so on. In the year 2020, the average life expectancy of registered residents in Beijing was 82.43 years. In 2020 there were 4.299 million registered elderly residents (aged 60 and above) in Beijing, accounting for 19.6% of registered residents. These numbers keep increasing; it is expected that by 2025, this figure will be close to 24%. The distribution of the elderly population in Beijing is uneven. In the central urban area, the aging population is the highest, with 65% of the elderly population in Beijing living in the six central urban districts (Dongcheng, Xicheng, Chaoyang, Haidian, Fengtai, and Shijingshan District).

The consequent pension problem has become an urgent social problem. Given China’s traditional culture, most elderly residents tend to live in their own homes rather than going to the elder nursing institutions. Therefore, the government tried to put forward the mode of combining home-based, community-based, and institutional care, and increased government investment and policy support for the elderly. The Elderly-Care Inns, as the main functional unit of elderly care service, have been widely established in urban communities in Beijing, as well as other big cities in China, aiming to provide home-based elderly care services for the surrounding community residents: meal, assistant, medical aid, day care, full care services, etc.

This home-based pension service mode is suitable to be the main pension model in China’s big cities. By the end of 2017, Beijing had established 380 community Elderly-Care Inns in the urban area. These Elderly-Care Inns have greatly alleviated the increasingly urgent demand for urban elderly care in Beijing, however, as a new thing, there is not accurate survey data about the pension needs, population characteristics, and health conditions of the urban elderly in China.

Thus, our study was designed to develop a survey among the elder population who lived in Beijing urban area for at least 5 years, and all received the service from the Elderly-Care Inns, to investigate the prevalence of osteoporosis, and health status of these population, in order to provide assistance for the construction of the hardware and the service contents, as well to provide data support and theoretical guidance for the combination of medical care and rehabilitation for the Elderly-Care institutions.

### Identification of High-Risk Population for Osteoporosis in the Elderly Urban Residents in Elderly-Care Inns in Beijing

In this study, a cost-effective method to evaluate the risk of abnormal BMD level (including osteoporosis and osteopenia) was established, based on hands grip strength, height loss, and familial Alzheimer’s history in men; and hands grip strength, height loss, history of fracture and steatohepatitis, and menstrual history in women. The predictive ability for osteoporosis is desirable in both men and women.

Hand grip strength was independently associated with increased fall risk score in osteoporotic elderly women (29), and low hand-grip strength was specifically associated with the risk of distal forearm fractures within 10 years and clinical vertebral fractures within 15 years or more in Japanese postmenopausal women (30). Since hand grip strength is easy and cost-effective to measure, it is suitable to be used as an easy assessment method for identifying individuals at a high risk of osteoporosis. The cutoff strength for evaluating osteoporosis in adults is age and sex specific (31). The results in this study were consistent with the previous studies.

Height loss is also simple to evaluate in the clinical setting, and it is a frequent manifestation of vertebral osteoporosis or fracture. The degree of height loss varied among individuals, and excessive height loss, of 3-4cm or more can be considered a simple indicator of increasing fracture risk (32, 33). In our study, height loss over 3cm was an independent risk factor for osteoporosis in both men (OR =1.613 and 95% CI= 1.028~2.527) and women (OR = 1.316 and 95% CI =1.042~1.786), which was consistent with the previous studies, in which the magnitude of the association translated to a 19% increase in odds for 1/2 in. and 177% for 3 in (34). Thus, height loss was suggested as routine evaluation in the outpatient setting for its ability in detecting osteoporosis of the hip (32, 35).

Steatohepatitis history was negatively associated with the risk of osteoporosis based on this study (OR = 0.677 and 95% CI =0.498~0.922). There existed controversy regarding the relationship between BMD and nonalcoholic fatty liver disease (36–38). As study concluded that the incidence of osteoporosis was significantly higher in the nonalcoholic fatty liver disease group (39). In addition, contradictory results have also been reported regarding a relationship between serum lipid levels and BMD. Some researchers believed that serum lipid levels could be potentially useful indicators to reflect the process of osteoporosis (40), some think that hyperlipidemia was associated with decreased BMD (41), and some found serum lipids did not directly and correlatively influence BMD (42, 43). Given that in the present study, we did not distinguish between alcoholic fatty liver and nonalcoholic fatty liver, and the serum lipid levels were not available, further studies are needed to clarify the relationship between steatohepatitis history or lipid parameters and osteoporosis.

In men, the cost-effective method adopts both hands grip strength, height loss over 3 cm, and familial Alzheimer’s disease history indicated a good predictive ability to evaluate the risk of osteoporosis (AUC=0.730, 95%CI=0.642~0.817) and abnormal BMD (AUC=0.672, 95%CI=0.600~0.743). In women, the method adopted both hands grip strength, height loss over 3 cm, Steatohepatitis history, and menstrual history (age of menarche, age of menopause, and duration of menstruation) achieved similar predictive ability (AUC=0.769, 95%CI=0.724~0.813).

The predictive abilities of serum testing variables, including serum levels of β-CTx, OST, and PINP in men and PTH and ALT in women, were close to those of the cost-effective method. β-CTx and PINP were designated as reference bone turnover markers in osteoporosis by the International Osteoporosis Foundation (IOF) and International Federation of Clinical Chemistry and Laboratory Medicine (IFCC) (44). The IOF-IFCC Joint Working Group on Bone Marker Standards (WG-BMS) recommended PINP and β-CTX be used as blood reference markers for bone formation and bone resorption, respectively, in osteoporosis (45, 46). Serum β-CTx and PINP distributed abnormally and stably in healthy men and women of Han nationality, the serum β-CTx and PINP reference interval were provided by several studies (47, 48).

Although the combination predictor including both cost-effective and serum testing factors achieved better predictive ability (in men AUC=0.797 with 95%CI=0.715~0.879; in women AUC=0.826 with 95%CI=0.787~0.864), considering the simplicity and accessibility of operation, the cost-effective factors were strongly recommended in the identification of high risk subjects in the elderly urban residents in Elderly-Care Inns in Beijing.

### Limitations and Prospective

There were still some limitations in this study. Firstly, the number of male subjects was relatively small, which might weaken the power of the conclusion. Secondly, there was too much missing data of some important indexes, such as the use of vitamin D and calcium, physical activity, and the determination of TCM symptoms and constitution, which made it difficult to conduct further in-depth analysis in the risk factor screening. In addition, as a cross-sectional study, the evidence that this study could provide was relatively limited, so long-term follow-up of this population is necessary to provide more evidence.

## Conclusion

In the population of elderly urban residents in Elderly-Care Inns in Beijing, the prevalence of osteoporosis in women was higher than that in men, and increased with aging more rapidly; yet, the prevalence of osteopenia in men was higher than that in women. After adjustment of age and BMI, both hands grip strength, height loss over 3 cm, serum levels of β-CTx, PINP, and OST were the independent risk factors for osteoporosis in both men and women; besides, familial Alzheimer’s disease history in men; and history of steatohepatitis and fracture, serum levels of PTH and ALT, age of menarche, age of menopause, duration of menstruation in women, were also risk factors of osteoporosis. The cost-effective method, including both hands grip strength, height loss over 3 cm, and familial Alzheimer’s disease history in men; fracture and steatohepatitis history and menstrual history in women, indicated good predictive ability to evaluate the risk of osteoporosis, thus was recommended in identifying the high risk subjects for osteoporosis.

## Data Availability Statement

The raw data supporting the conclusions of this article will be made available by the authors, without undue reservation.

## Ethics Statement

The studies involving human participants were reviewed and approved by The Medical Ethics Committee of Dongzhimen Hospital, Beijing University of Chinese Medicine. The patients/participants provided their written informed consent to participate in this study.

## Author Contributions

XL designed the study protocol and drafted the manuscript; HG, YL, JK, GW, and YC developed the questionnaires and data collection; JW and XH performed the data analysis; MJ and QY took part in the design of the study and MJ made revision of the manuscript. All authors contributed to the article and approved the submitted version.

## Funding

This research was supported by the National Clinical Research Base of TCM Project (JDZX2015079), the Scientific and Technological Innovation Project of China Academy of Chinese Medical Sciences (CI2021A05404), and the National Natural Science Foundation of China (81873181).

## Conflict of Interest

The authors declare that the research was conducted in the absence of any commercial or financial relationships that could be construed as a potential conflict of interest.

## Publisher’s Note

All claims expressed in this article are solely those of the authors and do not necessarily represent those of their affiliated organizations, or those of the publisher, the editors and the reviewers. Any product that may be evaluated in this article, or claim that may be made by its manufacturer, is not guaranteed or endorsed by the publisher.
